# *Bacillus pumilus* induced tolerance of Maize (*Zea mays L*.) against Cadmium (Cd) stress

**DOI:** 10.1038/s41598-021-96786-7

**Published:** 2021-08-25

**Authors:** Asim Shahzad, Mingzhou Qin, Mahmood Elahie, Muhammad Naeem, Tasmia Bashir, Humaira Yasmin, Muhammad Younas, Ahsan Areeb, Muhammad Irfan, Motsim Billah, Abdul Shakoor, Saman Zulfiqar

**Affiliations:** 1 College of Geography and Environment, Henan University, Jinming Ave, Kaifeng, China; 2 Department of Botany, Mohi-Ud-Din Islamic University, AJ&K, Pakistan; 3 Faculty of Life Sciences, Department of Biology, University of Okara, Okara, Pakistan; 4 Department of Plant Sciences, Quaid-I-Azam University, Islamabad, Pakistan; 5 Department of Biosciences, COMSATS University, Islamabad, Pakistan; 6 Department of Biotechnology, Mohi-Ud-Din Islamic University, AJ&K, Pakistan; 7 Department of Agronomy, Faculty of Agricultural Sciences and Technology, Bahauddin Zakariya University, Multan, Pakistan; 8 Department of Health Sciences, Abasyin University Islamabad, Islamabad, Pakistan; 9 Govt Sadiq College, Women University, Bahawalpur, Pakistan

**Keywords:** Biological techniques, Microbiology, Physiology, Plant sciences, Environmental sciences

## Abstract

Heavy metals contaminate the soil that alters the properties of soil and negatively affect plants growth. Using microorganism and plant can remove these pollutants from soil. The present investigation was designed to evaluate the induced effect of *Bacillus pumilus* on maize plant in Cadmium (Cd) contaminated soil. Three different concentrations of Cd (i.e. 0.25, 0.50 and 0.75 mg kg^−1^) were applied in soil under which maize plants were grown. The germination percentage, shoot length, leaf length, number of leaves, root length, fresh weight and nutrient uptake by maize plant were determined. The experiment was conducted by using complete randomized design (CRD) with three replicates. The result indicated that germination percentage, Shoot length, leaf length, root length, number of leaves, and plant fresh weight were reduced by 37, 39, 39, 32 and 59% respectively at 0.75 mg kg^−1^ of CdSO_4_ concentration but when maize seeds inoculated with *Bacillus pumilus* significantly increased the germination percentage, shoot length, leaf length, number of leaves, plant fresh weight at different concentrations of CdSO_4_. Moreover, the plant protein were significantly increased by 60% in T6 (0.25 mg kg^−1^ of CdSO_4_ + inoculated seed) and Peroxidase dismutase (POD) was also significantly higher by 346% in T6 (0.25 mg kg^−1^ of CdSO_4_ + inoculated seed), however, the Superoxide dismutase (SOD) was significantly higher in T5 (0.75 mg kg^−1^ of CdSO_4_ + uninoculated seed) and was 769% higher as compared to control. The Cd contents in *Bacillus pumilus* inoculated maize roots and shoots were decreased. The present investigations indicated that the inoculation of maize plant with *Bacillus pumilus* can help maize plants to withstand Cd stress but higher concentration of Cd can harm the plant. The *Bacillus pumilus* has good potential to remediate Cd from soil, and also have potential to reduce the phyto availability and toxicity of Cd.

## Introduction

Soil is composed of different components of solid, liquid and gases in the form of “organic, inorganic and mineral particles” which support plants and animals by providing or transferring energy in various ways in the environment^1^. These resources are divided into two main groups i.e. renewable and non-renewable resources. Soil is non-renewable resource, which is formed due to weathering of rocks by fluctuations in climate, weather and organismic activities^2^. At present time one of the main causes of pollution is heavy metals. These heavy metals remain untreated in soil and produce soil contamination which is very toxic for organisms. Heavy metals cannot be degraded by any process but it can be changed to less poisonous form^3^. These heavy metals cause various diseases to plants and animals by oxidative stress, their sources may be anthropogenic as well as natural^4^. Heavy metals pollution are foremost pollutant of our food particularly vegetables which is contaminated by absorbing heavy metals from polluted soil, water and air due to disposal of industries and urban waste. The elements that have density greater than 5 g cm^−3^ are called heavy metals^5^. The ingestion of heavy metals contaminated vegetables may lead to various long term lingering diseases like semphysema, bronchiolitis, and alveolitis, also short term disease like nervous, kidney, cardiovascular, and bone diseases^6^. Soil adulteration with heavy metals is a common problem for world which is alarming threat for human health^7^.

Cd is an unnecessary and greatly noxious heavy metal, which present in environment due to anthropogenic activities. Cd inhibit the plant to absorb important nutrients, in result plant growth is reduced which indicates Cd phytotoxicity^8^. Cd is non-amphoteric in nature and not properly dissolves in base solution^9^. The development of plant organs bears harmful effect of heavy metals like lead (Pb) and Cd which reduce biomass of various plant species^10^.

The plant species grown in contaminated soil having high concentration of pollutant reduce plant organ formation^11^. The crop which are produced in contaminated soil, absorb contaminants in their tissues and are very toxic for living organisms when are used as food^12^. Different plant species accumulate different types of heavy metals in their tissues from contaminate site^13^. Industrial pollutants contaminate water and play harmful impact on organisms. Uptake of toxic metals in plants effects variations in plant species, plants growth stage and translocation of metals^14^. These heavy metals damage molecular structure of plant and animals^15^. To eliminate contamination of non-degraded partials, phytoextraction is used which increase biomass and bio-concentration of plants^16^. There are different types of technologies used in present time to eliminate contaminants from polluted areas to reestablish natural condition. Phytoremediation is one of the best technology in which plant absorbs toxic substances from soil and water. Only selected plants are utilized for this purpose^16^. Phytoremediation is an ecofriendly technology to remove toxic metals^17^.

Numerous bacterial species are known that play vital role to tolerate plants under stress condition which can detoxify, transfer and collect heavy metals. Microorganisms and plants combine together against toxic effect of heavy metals by using rhizoremediation and phytoremediation mechanism. Microbes enhance the growth of plant in heavy metals stress^18^. Plant absorbs heavy metals in soil and transport from root to shoot via xylem tissue after physiological process accumulates into grains. Plants having different genotype and capacity to detoxify heavy metals stress^19^. Plant microbe’s interaction decomposes various pollutants and increase plant development and growth^20^. A bulk of enzymes from bacteria, have been reported to be concerned in the biodegradation of toxic organic pollutants and remove the soil contamination^21^.

Previous reports demonstrated that several species of *Bacillus* can beneficially promote growth and enzyme system which may help the plants to overcome the biotic stresses^22^. The application of several *Bacillus* strains in soil contaminated with heavy metals soil can help to reduce the harmful effects of heavy metals and enhances the plant growth. The *Bacillus* spp also have ability to accelerate the plant growth by increasing water uptake and reducing electrolyte leakage to mitigate Cd stress^23^. *B. licheniformis* enhances Cu, Zn, Cd, Cr and Pb accumulation and distribution in plants grown in heavy metal-contaminated soil, which leads to reduced levels of toxic metals in soil^24^. Similarly, higher concentration of Cd in soil reduce nutrient (P, Fe, Zn, and Mn) uptake in plants. *B. pumilus* is a promising plant growth promoting bacteria and previous reports ^25^ demonstrated that *B. pumilus* affected metal toxicity in tomato and rapeseed (*Brassica napus* L.) The application of *Bacillus spp.* alleviate stress effect by reducing lipid peroxidation and SOD activity and increasing amylase and protease to promote plant growth in heavy metal-polluted soil^26^. Similarly, *Bacillus spp.* support plant tolerance against Zn and Cu stress by enhancing the activities of ROS scavenging enzymes, such as POD, SOD, CAT, APX, and DHAR^27^. The regulation of antioxidants in cells inhibits oxidative stress damage and triggers plant growth-promoting substances to enable plants to adapt to metal stress. Bacillus-mediated plant tolerance against Ni and Cr stresses is achieved through the enhancement of photosynthetic pigments and leghemoglobin, which leads to increased crop yield^28^. However, the effect of *B. pumilus* on Cd uptake by plants has received lesser attention. It is not clear whether plant physiological processes work independently or together with other mechanism like antioxidant system of plant under cd stress. In this context, the present study was therefore performed to investigate the potential of *Bacillus pumilus* to induce growth and antioxidant enzymes of maize plants under Cd stress.

## Materials and method

### Preparation of heavy metal solution

Three different concentration of cadmium sulphate (CdSO_4_) solution (0.25, 0.50 and 0.75 mg mL^−1^) were prepared for different treatment in pure distilled water by dissolving the cadmium sulfate (CdSO_4_). The different concentrations of CdSO_4_ were selected on the basis of the previous scientific data^29^. Pure distilled water was used as control for the experiment. 100 mL of each solution was added in 1 kg of potted soil.

### Preparation of bacterial inoculum

The *Bacillus Pumilus* (Acc KF859972) used in this study was taken from phytohormone Lab Quaid-i-Azam University,Islamabad, Pakistan, on the basis of its plant growth indorsing latent^30^**.** For the preparation of inoculum, the nutrient broth was purchased from OXOID-UK. The nutrient broth was sterilized at 121 °C for 20 min. The isolated strain was inoculated in nutrient broth and incubated in shaker incubator (EXCELLA E24 Germany) at 150 rpm for 48–72 h. After that, the culture was centrifuged for 10 min at 3000 rpm**.** The pellet was again suspended in double distilled water and optical density (O.D) was adjusted to 0.100 at 660 nm with UV–VIS spectrophotometer. The inoculum was prepared by culture of bacterial strain having O.D 0.100 at 660 nm and bacterial density (10^6^ cells/ml).

### Seed inoculation

Maize (*Zea mays* L.) seeds (KASHMIR GOLD) was obtained from NARC (National Agricultural Research Centre) Islamabad, Pakistan. The seeds were washed with ethanol (95%) for surface sterilization, following by soaking in 10% Chlorox for 2–3 min and subsequently the seeds were washed successively 2–3 times with autoclaved distilled water^31^. Moreover all the methods were performed in accordance with the relevant guidelines given by the national agriculture research center for the cultivation of maize plants.

### Preparation of treatment applications

The seeds were dipped in the inoculum for two to 2–3 h. Then three different solutions of Cd sulphate prepared (i.e. 0.25, 0.50 and 0.75 mg kg^−1^). Eight different treatments with three replicates **(three Pots per treatment)** were made and five seeds of maize were sown in each pot (Table 1). For further analysis plants were harvested after 28 days of sowing.

**Table 1:** Preparation of treatment applications.

Treatments	*Bacillus pumilus* inoculation	CdSO_4_ (mg kg^−1^)
T1	−	0
T2	+	0
T3	+	0.25
T4	+	0.50
T5	+	0.75
T6	−	0.25
T7	−	0.50
T8	−	0.75

### Parameter measured

The germination percentage was observed after four day of sowing whereas, maize were harvested after 28 day of sowing. In order to remove non-aggregated soil, seedlings were slightly shaken. The following parameters were studied^32^. Shoot and root lengths were measured from the root initiation up to the tip of the longest shoot and root. It was measured in centimeters^33^. Leaf size was measured in cm, from node to tip of the leaf^34^. Root length was measured from the junction of root and stem towards the tip of the longest root. It was measured in centimeters^35^. After harvesting plants from the pots, they were shaken to remove extra soil other than aggregates, the weight measured in grams^36^. Each plant of each replicate was measured and their mean value was used to compare the treatments.

### Leaf proline

Proline content of maize plant leaves was determined by the method of^37^ about 0.5 g of fresh maize leaves were used to determine the proline content.

$${\text{Proline}}\,{ = }K \, \times {\text{ Dilution factor }} \times {\text{ optical density/weight of sample}}$$

where the *K* value is 19.6.

### Peroxidase dismutase assay

The POD activity of maize leaves was measured by the method of^38^ about 1 g of fresh maize leaves were used to determine the POD enzyme.

### Superoxide dismutase assay

The SOD activity of maize leaves was measured by the method of^39^. The activity of SOD was expressed as units/100 g fresh weight. About 0.2 g of fresh maize leaves were used to determine the SOD enzyme.

### Plant nutrient analysis

The per chloric-acid digestion method was used to determined presence of the nutrients in the plant organs like root leaves and shoot^40^. Maize leaves (0.25 g) were used for nutrient analysis.

- “Cations in plants= (ppm in extract − blank) × A × dilution factor”
- “WA = Total volume of extract (mL)”.
- “W = Weight of dry plants.

### Statistical analysis

The experiment was conducted in a completely randomized design (CRD) by using Statistic 8.1.1. (https://statistix.informer.com/8.1/).The results are the compare means and standard error of means of three replicates of a treatment.

## Results

The experiment was carried out in pots with complete randomize design (CRD) and plants were harvested after 28th day of seed sowing and results were analyzed. Different parameter were observed i.e. fresh biomass, root length, shoot length, leaf size and number of leaves, Cd contents in roots, shoots and seeds germination .

### Effect of cadmium (Cd) on maize seed germination percentage

**Figure 1:**
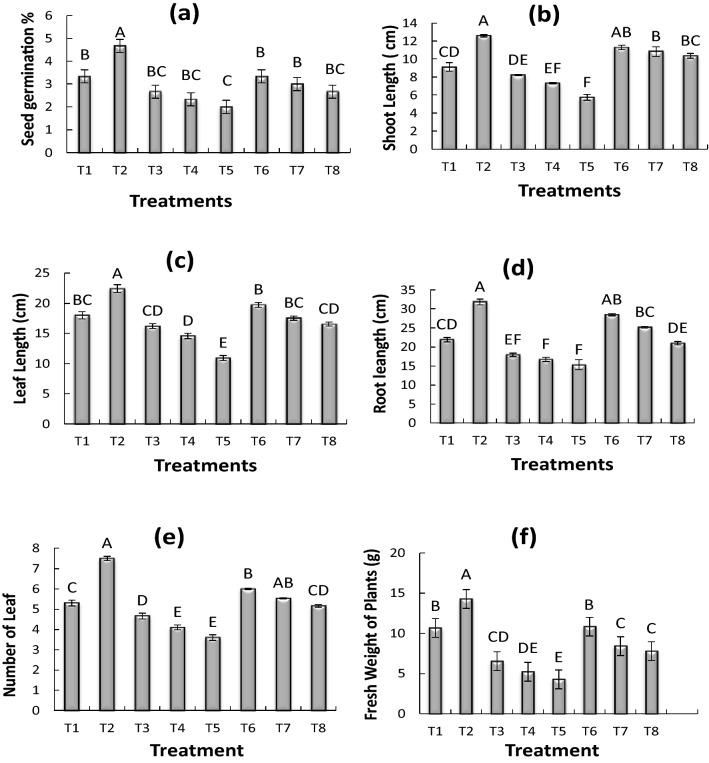
Effect of Cadmium (Cd) on Maize Seed Germination % (**a**), shoot length (**b**), leaf length (**c**), root length (**d**), no. of leaves (**e**), fresh weight (**f**). All treatments sharing common letter with similar bar pattern are similar otherwise differ significantly at *p* < 0.05. T1 = control, T2 = inoculated seed, T3 = 0.25 mg CdSO_4_ 100 mL^−1^ + uninoculated seed, T4 = B = 0.50 mg CdSO_4_ 100 mL^−1^ + uninoculated seed, T5 = 0.75 mg CdSO_4_ 100 mL^−1^ + uninoculated seed, T6 = 0.25 mg CdSO_4_ 100 mL^−1^ + Inoculated seed, T7 = 0. CdSO_4_ 100 mL^−1^ + Inoculated seed, T8 = 0.75 mg CdSO_4_ 100 mL^−1^ + Inoculated seed.

The germination percentage was significantly increased with the inoculation of *Bacillus pumillus* (T2), however the inhibition in germination was observed at all concentration of Cd as compared to the control. About 39% reduction in seed germination percentage was observed in T5 (0.75 mg kg^−1^ CdSO_4_ + uninoculated seed) as compared to control. While inoculation of *Bacillus pumillus* in the presence of Cd increased the germination percentage however this increase was non-significant (Fig. 1a). The germination percentage was increased in *Bacillus pumillus* inoculated seeds as compared to control and uninoculated seeds. The maximum seed germination was observed in T2 (*Bacillus pumillus* inoculated seed) which was 40% higher than control.

### Effect of cadmium (Cd) on maize shoot length (cm)

The *Bacillus pumilus* inoculation (T2) significantly induced shoot length of maize plant as compared to control (T1) but cadmium (Cd) inhibited the shoot length and maximum reduction (37%) in shoot length was observed in (T5) however *Bacillus pumilus* inoculation significantly increased the shoot length (Fig. 1b). The maximum shoot length was observed in treatment T2 (*Bacillus pumilus* inoculated Seeds.) which was 39% higher than control while 37% reduction in shoot length was observed in T5 (0.75 mg CdSO_4_ kg^−1^ + uninoculated seed) as compared to control. Cd also affected leaf length of maize plants. However, the inoculation of maize seeds with *B. pumillus* significantly enhanced the leaf length of maize plant at different concentration of Cd as compared to control and uninoculated maize plants. Moreover, 40% increase in leaf length was observed in T2 (*Bacillus pumillus* inoculated seed) as compared to control while, seeds showed 39% reduction in leaf length in T5 (0.75 mg CdSO_4_ kg^−1^ + uninoculated seed) as compared to control (Fig. 1c). The Fig. 1d shows the root length of maize plant affected by Cd ; however, the root length of inoculated maize seeds with *Bacillus pumilus* significantly increased when grown at different concentrations of Cd . The maximum (40%) root length was observed in T2 (*Bacillus pumilus* + seed) which were 40% higher than control and uninoculated plants, as compared to control. The reduction in root length was observed at different concentration of Cd and about 39% reduction in root length was observed in T5 (0.75 mg CdSO_4_ kg^−1^ + uninoculated seed) as compared to control, however inoculation of *Bacillus pumilus* significantly enhanced the rood length at different concentrations of Cd. The Cd affected the number of leaves in maize plants (Fig. 1e), while inoculation of maize seeds with *Bacillus pumilus* enhanced the number of leaves in maize plants at different concentrations of Cd. The number of leaves were increased by 42% in T2 (*Bacillus pumilus* inoculated Seed) as compared to control and uninoculated seeds, while uninoculated seeds T5 (0.75 mg CdSO_4_ kg^−1^ + uninoculated seed) showed 32% reduction in number of leaves as compared to control. The result presented in Fig. 1 shows the Cd affected fresh weight of maize plants. However, inoculation of maize seeds with *Bacillus pumilus* notably enhanced the fresh biomass of maize plant at different concentration of Cd. The maximum (34%) plant fresh weight was observed in T2 (*Bacillus pumilus* inoculated seed) as compared to control, while 59% reduction in plant fresh biomass was observed in T5 (0.75 mg CdSO_4_ kg^−1^ + uninoculated seed) as compared to control.

### Effect of cadmium (Cd) on plant protein

**Figure 2:**
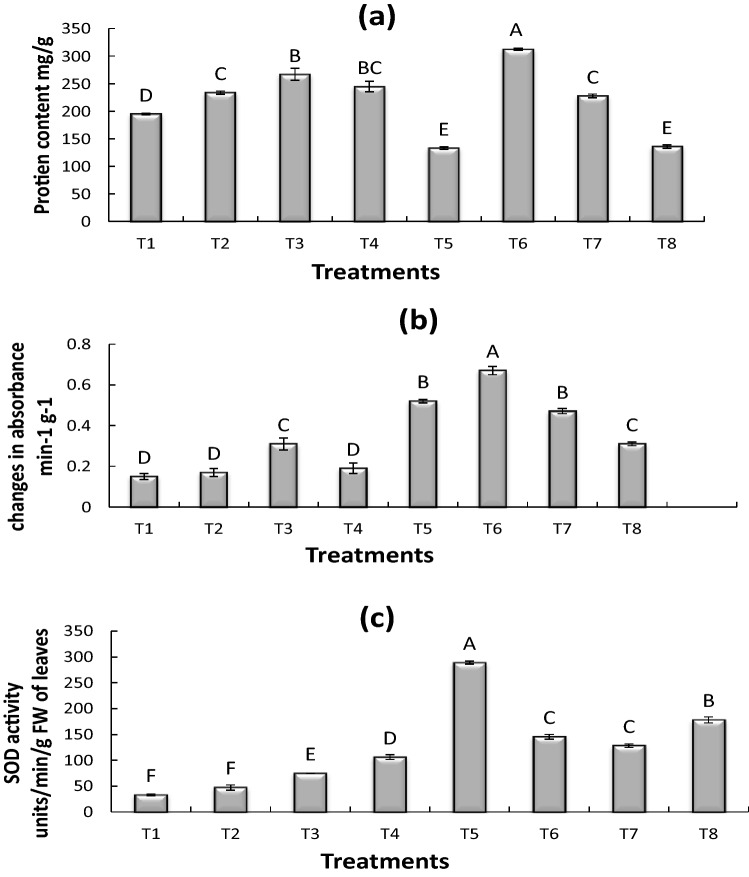
Effect of Cadmium (Cd) on maize protein (**a**), antioxidant enzymes peroxidase dismutase (**b**) and super oxidase dismutase (**c**). All treatments sharing common letter with similar bar pattern are similar otherwise differ significantly at *p* < 0.05. T1 = control, T2 = inoculated seed, T3 = 0.25 mg CdSO_4_ 100 mL^−1^ + uninoculated seed, T4 = B = 0.50 mg CdSO_4_ 100 mL^−1^ + uninoculated seed, T5 = 0.75 mg CdSO_4_ 100 mL^−1^ + uninoculated seed, T6 = 0.25 mg CdSO_4_ 100 mL^−1^ + Inoculated seed, T7 = 0. CdSO_4_ 100 mL^−1^ + Inoculated seed, T8 = 0.75 mg CdSO_4_ 100 mL^−1^ + Inoculated seed.

Though a reduction in the protein content was observed in T5 (0.75 mg CdSO_4_ kg^−1^ + uninoculated seed), and T8 (0.75 mg CdSO_4_ kg^−1^ + inoculated seed) where heavy metals concentration was higher. But, the protein content percentage was significantly higher in T6 (0.25 mg CdSO_4_ kg^−1^ + inoculated seed) by 60%. However, the lower concentration of Cd in (Cd) T3 (0.25 mg CdSO_4_ kg^−1^ + uninoculated seed) also triggered the protein content in maize cultivar by 36% compared to control. In the presence of *Bacillus pumilus* T2 and T7 (0.50 mg CdSO_4_ kg^−1^ + inoculated seed), showed an increase of 36 and 16% (Fig. 2a).

### Effect of cadmium (Cd) on peroxide dismutase (POD) enzyme

To scrutinize the effect of various concentrations of Cd, the antioxidant activities (POD and SOD) were determined. Results exhibited that treatment T6 (0.25 mg CdSO_4_ kg^−1^ + inoculated seed), T5 (0.75 mg CdSO_4_ kg^−1^ + uninoculated seed), T7 (0.50 mg CdSO_4_ kg^−1^ + inoculated seed), T8 (0.75 mg CdSO_4_ kg^−1^ + inoculated seed), and T3 (0.25 mg CdSO_4_ kg^−1^ + un inoculated seed) showed a significant increase of 346, 246, 213, 106 and 106% as compared to control respectively. On the other hand, a reduction of 13% in POD activity was observed in treatment T2 (*Bacillus pumilus*). (Fig. 2b).

### Effect of cadmium (Cd) on superoxide dismutase (SOD) enzyme

The maximum antioxidant (SOD) activity was observed at a higher concentration of Cd specifically in T5 (0.75 mg CdSO_4_ kg^−1^ + uninoculated Seed). The significant percentage increase of SOD was 769% when compared with control. Likewise, compared with control all other treatments showed a significant increase of SOD enzymatic activity in the presence of *Bacillus pumilus* in T8 (0.75 mg CdSO_4_ kg^−1^ + inoculated Seed ), T6 0.25 mg CdSO_4_ kg^−1^ + inoculated seed), T7 (0.50 mg CdSO_4_ kg^−1^ + inoculated seed) and in uninoculated treatments; T4 (0.5 mg CdSO_4_ kg^−1^ + uninoculated Seed), T3 (0.25 mg CdSO_4_ kg^−1^ + uninoculated Seed) by 437, 338, 287, 220, and 125%. While treatment T2 (*Bacillus pumilus*) showed the least increase with 43% higher than control (T1) (Fig. 2c).

### Accumulation of cadmium (Cd) in maize roots (mg/g)

**Figure 3:**
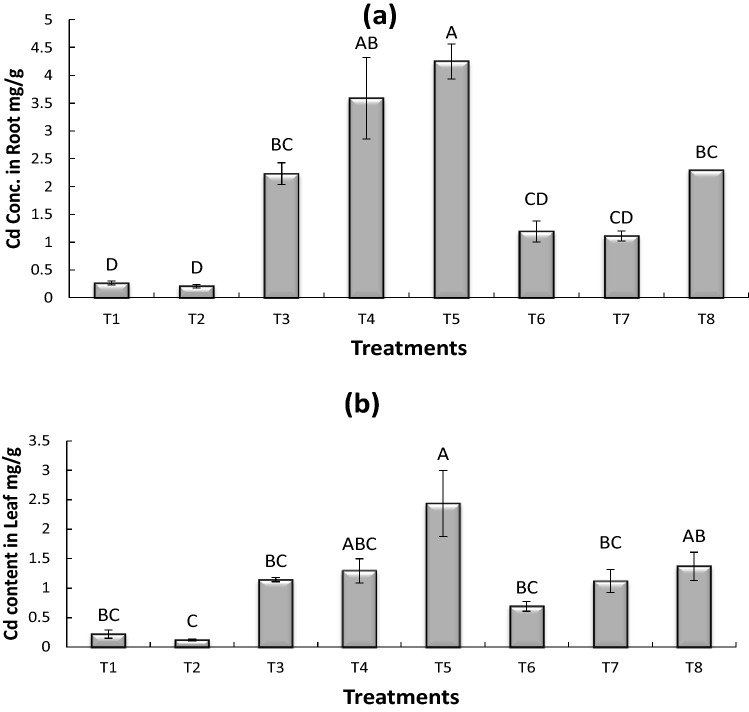
Accumulation of Cadmium (Cd) in maize roots (**a**) and maize leaves (**b**). All treatments sharing common letter with similar bar pattern are similar otherwise differ significantly at *p* < 0.05. T1 = control, T2 = inoculated seed, T3 = 0.25 mg CdSO_4_ 100 mL^−1^ + uninoculated seed, T4 = B = 0.50 mg CdSO_4_ 100 mL^−1^ + uninoculated seed, T5 = 0.75 mg CdSO_4_ 100 mL^−1^ + uninoculated seed, T6 = 0.25 mg CdSO_4_ 100 mL^−1^ + Inoculated seed, T7 = 0. CdSO_4_ 100 mL^−1^ + Inoculated seed, T8 = 0.75 mg CdSO_4_ 100 mL^−1^ + Inoculated seed.

There was variation in the accumulation of Cd contents in maize roots which was observed in all the treatment as shown in Fig. 3a. The maximum 45% Cd uptake was found in T5 (0.75 mg CdSO0_4_ kg^−1^ + uninoculated Seed) as compared to control. However, the Cd accumulation was reduced in all treatments when inoculated with *Bacillus pumilus* as compared to uninoculated seeds. The minimum 21% Cd contents in maize plant were observed in T6 (0.25 mg CdSO0_4_ kg^−1^ + inoculated Seed).

### Accumulation of cadmium (Cd) by maize leaves (mg/g)

The Fig. 3b showed variation in Cd contents in maize plant leaves in all the treatment, however the maximum 90% Cd uptake was found in T5 (0.75 mg CdSO0_4_ kg^−1^ + uninoculated Seed) as compared to control. While Cd concentration was reduced in all treatments when inoculated with *Bacillus pumilus* as compared to control and uninoculated seeds. (The minimum 45% Cd contents in maize plants observed in T6 (0.25 mg CdSO0_4_ kg^−1^ + inoculated Seed).

### Accumulation of micro and macro nutrients by maize plants

The results presented in Table 2 showed Cu content increased in all the treatments when inoculated with *Bacillus pumilus* as compared to uninoculated plants. The maximum Cu content (10.14C) was observed in T3 and minimum in T5 (0.2D). The Mn content showed variations in inoculated and uninoculated seeds, and maximum content of Mn (7.9A) was recorded in T3 while minimum content of Mn was observed in T7 (1.16D). Inoculation decreases Na content in plant as compared to uninoculated plants. Maximum Na content observed in T3 (6.11A) and minimum was reported in T8 (0.48E) Fe content showed variation in inoculated seeds as compared to uninoculated seeds. Maximum Fe content was reported in T1 (2.89A) while minimum Fe was in T7 (0.54D). Inoculation of *Bacillus pumilus* increases Ca content in plants while uninoculated plants have low Ca content. Maximum Ca content found in T3 (6.81A) and minimum were found in T8 (0.60E) Mg and K content also showed variation in inoculated and uninoculated plants. Maximum Mg concentration was observed in T2 (1.36A) while minimum concentration was recorded in T5 (0.62D), and maximum K content was noted in T2 (2.72A) and minimum in T8 (o.29D).

**Table 2:** Accumulation of micro and macro nutrients by maize plants.

Nutrients	Nutrient concentration							
	T1	T2	T3	T4	T5	T6	T7	T8
Cu (mg/g)	4.33 ± 0.0.35C	6.84 ± 0.171B	4.33 ± 0.37C	2.66 ± 0.151D	1.98 ± 0.13BD	2.66 ± 0.29CD	2.37 ± 0.28D	2.04 ± 0.26D
Mn (mg/g)	3.28 ± 0.016C	6.40 ± 0.26B	10.47 ± 0.22A	3.33 ± 0.07C	1.78 ± 0.07BD	1.62 ± 0.18D	1.16 ± 0.04D	1.62 ± 0.21D
Na (g/Kg)	1.57 ± 0.03D	5.13 ± 0.18B	6.11 ± 1.21A	2.56 ± 0.19C	2.27 ± 0.17C	0.90 ± 0.04E	0.896 ± 3.03E	0.49 ± 0.5E
K ( mg/g)	2.62 ± 0.14A	2.72 ± 0.15A	1.44 ± 0.03B	1.03 ± 0.01BC	0.38 ± 0.01D	0.41 ± 0.02D	0.83 ± 0.22CD	0.29 ± 0.02 D
Fe (mg/g)	2.89 ± 0.24A	1.65 ± 0.15BC	1.41 ± 0.15BCD	0.70 ± 0.14BCD	1.15 ± 2.26BCD	2.04 ± 8.23AB	0.54 ± 1.19D	1.29 ± 0.20BCD
Ca (g/Kg)	1.68 ± 0.12D	4.53 ± 0.14B	6.81 ± 0.13A	5.26 ± 0.020B	2.49 ± 0.13C	2.36 ± 0.19DCD	2.47 ± 0.20C	O6.07 ± 0.14E
Mg (g/Kg)	1.18 ± 0.01AB	1.36 ± 0.142A	0.75 ± 0.13BCD	1.11. ± 0.020ABC	O.62 ± 0.019CD	1.066 ± 0.020ABCD	0,56 ± 0.0135D	0.66 ± 0.54CD

All treatments sharing common letter are similar otherwise differ significantly at p < 0.05. T1 = control, T2 = inoculated seed, T3 = 0.25 mg CdSO_4_ 100 mL ^−1^ + uninoculated seed, T4 = 0.50 mg CdSO_4_ 100 mL ^−1^ + uninoculated seed, T5 = 0.75 mg CdSO_4_ 100 mL^−1^ + uninoculated seed, T6 = 0.25 mg CdSO_4_ 100 mL^−1^, + Inoculated seed, T7 = 0. CdSO_4_ 100 mL^−1^ + Inoculated seed, T8 = 0.75 mg CdSO_4_ 100 mL^−1^ + Inoculated seed.

## Discussion

The Cd contaminant adversely affects plants and animals directly and indirectly however, trace amount of Cd in soil did not harm plants^12^. Cd enters into soil in different anthropogenic activities as well as by natural process. Heavy metals present in soil and air remain untreated and enters plant body through dust and moisture contents, which first impacts seeds, and roots of plants, afterwards damages shoots and leaves respectively^41^. It is obvious that germination or growth of plants is increased in inoculated treatments and the growth of maize plant affected by high concentration of Cd, however the effect is minimized by inoculating with *Bacillus pumilus*. During the present study, the germination was improved with the inoculation of *Bacillus pumilus* when grown over Cd and these findings are in agreement with^42^, who reported that the inoculation of plant seeds with microorganism species like *Pseudomonas, Pasteurella, Salmonella, Bacillus* and *Burkholderia* have the ability to resist. The result finding are also supported by^43^, who reported that Cd toxicity has decreased seed germination percentage.

The removal of heavy metals contaminants from contaminated site, the combined application of plant and microbe is a successful method as compared to the use of plant or bacteria separately^44^. The higher concentration of lead (Pb) reduces the flower production^45^. In the present study, Cd affected the maize plant in the same way. The higher concentration of Cd can cause plant toxicity and reduction in growth through interference with mineral and Cd absorption, and movement of necessary elements^8^, The findings of present study are in accordance with these results. The Cd concentration reduced the plant growth and prompted phytochelatin (PC), Cd destructively lowers plant growth because it is non-essential element^46^. Inoculation of seeds with *Bacillus pumilus* also enhanced plant growth, this increase in plant length might be due to the production of phytohormones^47^. It is also reported that the growth in bacterial inoculated seeds with different Cd concentrations showed significant leaf growth, which showed that bacterial inoculation can promote the tolerant capacity of plants which are in agreement with our findings in which the seed inoculated with *Bacillus pumilus* showed better leaf growth under Cd stress^48^. Cd transported from soil to all parts of plants tissue, damages the tissues in various ways, so size of contaminated leaf stunted. Likewise^49^, reported visual symptoms of chlorosis and necrosis in tomato plant when applied up to 25 and 50 μM of CdCl2. We also got same result when 75 mg dose of Cd on maize plant caused wilting in uninoculated treatment but inoculated treatment did not showed these symptoms because *Bacillus pumilus* inhibit toxic symptoms by providing tolerance ability.

Root is the first organ of plant which is affected by Cd and Cd adversely affects the root length. The study of^50^, showed similar findings which showed decreased root length in the presence of Cd without any inoculation, because Cd destroyed the protein structure however root length showed better growth when inoculated with *Bacillus pumilus* and accumulation of Cd in roots of *Barlay* plant was 25% more than stem which inhibited the normal growth of plant root^51,52^. The effects of heavy metals depend on type of environment and toxic substances uptake by plants. Greater the toxic substance in soil will cause reduction in plants growth^53^ also confirmed our finding that in high level of Cd the maize plant showed reduced growth.

The Cd stress in maize plant produce free radicals which damage membrane and cause leakage of electrolyte^50^, therefore number of leaves decreased in Cd stress^54^ reported soybean plant change its physiology as well as morphology like number, shape and size of leaf against Cd is agreement of our present finding in which the inoculation *Bacillus pumilus* significantly change the structure of bacterial community which enhance growth as compare to control after 15 days of experiment^55^ confirmed our findings that *Bacillus pulmilus* promote the tolerance capacity of plants.

In this study *Bacillus pumilus* also enhanced plant fresh weight by producing phytohormones like IAA and GA^56^. These hormones increase the plant root and shoot length, and leaf volume which promote fresh weight of maize plant. The Bacillus species also responsible for bioavailability of macro and micro nutrients from soil^57^ have beneficial effect on plant fresh weight.

Root secretions have vital function in altering metal bioavailability, these secretions have various compounds that combine with metals and restrict their movement in soil. These rhizo secretions also provide essential elements to microbial communities that enhance their growth and survival ability. Root secretions have different enzymes and protons that make the soil acidic and increase the heavy metal bioavailability^58^.

Maize plant accumulate Cd in shoots and inhibit the growth of shoot by damaging cell membrane which remove ions from damage site Cd^59^. Result presented in this experiment shows that Cd uptake by maize plant decrease in all treatments that were inoculated with *Bacilus pumillus* as compared to control and stressed plants. The reduction in Cd uptake was observed in plants that were inoculated with *Bacillus pumilus* and highest Cd uptake was observed in uninoculated plants. *Bacillus pumilus* converts Cd in to unavailable form in soil, and also reduces its toxicity. Previous studies also supported these results that inoculation with *Bacilus* species reduces Cd bioavailability^60–62^.

The plant possessess a well-organized antioxidant defense system. The accumulation of Cd toxicity was observed in maize cultivar with various treatments with *B. pumillus* and without *B.pumillus* inoculation in order to discern their ability to tolerate different concentration levels of Cd. The present study revealed that antioxidant activities (POD and SOD) stimulated at the higher concentration of Cd. The higher Cd concentrations in maize cause an increase in enzymatic activities because of the activation of enzymes that are already present in plants^63–66^. Comparable changes in the enzymatic activities under different concentrations of heavy metals specifically Cd toxicity have been reported earlier^67,68^. However, some of the studies are in deviation with our results reporting a decrease in SOD activity under the higher concentration of Cd level ^69–71^. The deviation in results could ensue due to the difference in the time duration of Cd stress applied, the intensity of Cd, and specifically plant stage and cultivar. Moreover, no significant increase was observed in maize plants treated with *Bacillus pumilus*^72^.

Present study depicted an increased SOD and POD activity at higher concentrations suggesting that both of these enzymes act simultaneously to avert the formation of OH ions and remove H_2_O_2_^71,73^. Therefore, the increased enzymatic (particularly SOD) activity at a higher concentration of Cd is considered a good indication for defensive mechanism stimulation^74^. In addition to this, it was observed in a study that the SOD activity was higher at the lower concentration of Cd in soil (20–25 mg/kg), normal when the concentration ranges between 50 and 75 mg/kg Cd in the soil and start to decrease when the soil Cd toxicity levels reached to 100 mg/kg^71^. The decrease in the enzymatic activity perhaps might be attributed to inhibition caused by accelerating H_2_O_2_^75,76^. Thus, heavy metal stress causes an induction of SOD and POD enzymes which in return provides protection and membrane integrity.

It is a known phenomenon that Cd stress leads to the denaturation of proteins. The present study validated the phenomenon that with the gradual increase in the Cd toxicity level the protein content started to decrease. The results are in agreement with the preceding studies demonstrating the reduction of protein content in maize due to Cd stress^77–79^.

Heavy metals like aluminum, nickel, lead, and Cd accumulate in root of plants and effect metabolisms of plant by reducing cell elongation and new cell formation^80^, so plant cannot promote their growth. Similarly in our present study plants treated with Cd showed stunted growth and accumulates maximum Cd in their roots^81^ also reported that most plant species like cucumber, rice, maize and etc. hold chief Cd concentration in their roots which reduced the plant growth by disturbing their metabolic activity. Cd. Soil polluted with Cd impacts roots of plants directly which disturb roots to uptake essential nutrients for metabolic activities of plants. However different plant species have tolerance capacity against specific heavy metals^82^.

## Conclusion

In conclusion, Cd adversely affects the growth of maize (*Zea mays*) plant; however inoculation of maize seeds with *Bacillus pumilus* promoted the tolerance to Cd toxicity. The application of *Bacillus pumilus* (T2) showed significant affect than all other treatments in germination, plant height, leaf length, number of leaves and fresh weight. Higher Cd concentration in soil inhibited plant growth, while the inoculation of *Bacillus pumilus* significantly reduced the adverse effect of Cd in all the treatments. Treatment T6 was significantly different from all other treatments under Cd stress. Furthermore, the uptake of Cd in maize is decreased in the presence of *Bacillus pumilus* in soil which reduced the mobility of Cd leading to less Cd accumulation in maize plant. However 0.75 mg/100 ml of Cd was toxic to maize plant but the inoculation of maize seed with *Bacillus pumilus* was effective to reduce Cd toxicity and uptake (T5 and T8). The present investigation reveals that *Bacillus pumilus* inoculation can be used as bio-fertilizer in different level of Cd stress soil.
